# Perspectives of the Saudi medical students toward the impact of lockdowns on their physical activity level and lifestyle during the COVID-19 pandemic

**DOI:** 10.7717/peerj.14725

**Published:** 2023-01-20

**Authors:** Asma Alrushud, Dalyah Alamam, Muneera Almurdi, Ghaliah A. Dablan, Alanoud A. Alghamdi, Fatimah K. Almazyad, Malak A. Alhamdani, Khlood A. Alqarni, Hosam Alzahrani

**Affiliations:** 1 Department of Health Rehabilitation Sciences, College of Applied Medical Sciences, King Saud University, Riyadh, Saudi Arabia; 2 Department of Physical Therapy, College of Applied Medical Sciences, Taif University, Taif, Saudi Arabia

**Keywords:** Exercise, Well-being, COVID-19, Quarantine, Global health

## Abstract

**Background:**

Physical activity (PA) level is affected by various factors in university students. Due to the pandemic of COVID-19, the Saudi Ministry of Education announced the closure of schools and universities as a preventive measure. This cross-sectional study aimed to evaluate the impact of the COVID-19 pandemic lockdown on PA levels and other lifestyle aspects among the Saudi medical students and to explore their perspectives toward it.

**Methods:**

Three hundred ninety-six medical students have completed the survey, which consisted of three sections: (1) participant’s demographic data; (2) five statements about the PA and the lockdown; and (3) the International PA Questionnaire—Short Form (IPAQ-SF).

**Results:**

About 63.9% of the participants were female, and 60.4% were of normal weight. Approximately 80% of participants were classified as inactive. Regarding the perspective of the students, 52.8% reported that they were not exercising regularly in the gym before the lockdown, and 46.9% reported that the lockdown and transition to online learning affected their compliance with exercise. During the lockdown and shift to online learning, the majority of participants experienced decreased PA levels, 42.4% experienced weight gain, and 53.6% reported a negative impact on their psychological status.

**Conclusion:**

Generally, the results of this study showed that the COVID-19 had a negative influence on the PA level, as well as other lifestyle aspects (*e.g.*, gaining weight) and psychological status of medical students. This study highlights aspects where universities and institutions delivering medical education can use resources to improve students’ well-being during pandemics.

## Introduction

Physical activity (PA) is defined as any bodily movement that is performed by skeletal muscles and requires energy expenditure that is measured in kilocalories (World Health Organization, 2022). Engaging in PA promotes multiple benefits such as improving cardiorespiratory fitness and muscle movements, maintaining healthy bones and skeleton systems, and improving individuals’ mental ability and overall quality of life (AlTamimi et al., 2021; World Health Organization, 2022).

According to the World Health Organization (WHO) guidelines, adults are recommended to engage in at least 150–300 min of moderate-intensity aerobic PA or 75–150 min of vigorous-intensity PA, or a combination of both, throughout the week (World Health Organization, 2022). It has been reported that about 5.3 million deaths annually are directly related to the lack of PA (Wen & Wu, 2012). Furthermore, previous research found a significant association between physical inactivity and the development of various diseases including cancers, weight gain, cardiovascular disease, endocrine disease, and elevated levels of cholesterols and triglycerides (Alahmed & Lobelo, 2018; Stubbs et al., 2018; Joy, Vincent & Vincent, 2020; An et al., 2020; AlTamimi et al., 2021).

Globally, the level of PA reported among university students was low and insufficient (Rejali & Mostajeran, 2013; Janampa-Apaza et al., 2021). This may be due to some factors including smoking, overweight and, unhealthy eating habits, and a sedentary lifestyle (Jiang et al., 2019; Lavie et al., 2019). University students try to cope with the stress associated with their lifestyle and academic routine (Ruiz, Ontoso & Guillén-Grima, 2019; Bashatah, 2020; Samreen, Siddiqui & Mothana, 2020; Althumiri et al., 2021). However, in Saudi Arabia, university students are not active to meet the required guidelines for moderate PA (Al-Hazzaa, 2018).

Lifestyle has been defined by the Oxford English Dictionary as “the way in which one lives” (Veal, 1993). A healthy lifestyle means practicing a number of habits including regular PA, a balanced diet, and refraining from smoking and excessive alcohol consumption (World Health Organization, 1999).

A global pandemic was triggered by the 2019 coronavirus disease (COVID-19) (Barkley et al., 2020). It has reached most countries around the world. According to the WHO, since it is becoming increasingly difficult to stop the spread of the virus, countries must take strict steps to contain the pandemic (Gallo et al., 2020). Physical distance guidelines have altered social experiences during the pandemic, leading to the closure of non-essential businesses and public facilities such as gyms, outdoor parks, restaurants, and reduced opportunities for leisure time activities (Barkley et al., 2020). These measures lead to minimizing both PA frequency and duration, not only for traditionally active individuals who are no longer able to join gyms and health clubs but for those who incidentally obtain appropriate levels of activity by walking or cycling to work or study. This is especially relevant to university students attending a major campus who usually walk many times a day between classes (Gallo et al., 2020). When the world was battling with one of the biggest health crises caused by novel COVID-19 educational institutions around the world such as universities had to shift rapidly to online and distance learning (Almaiah, Al-Khasawneh & Althunibat, 2020).

The impact of the lockdown on PA levels due to COVID-19 was investigated in different populations around the world. For example, a previous study conducted among population in Italy showed that the PA level significantly decreased during and after the lockdown (Mauro et al., 2022). Moreover, another study conducted among young Kosovan population reported that the PA level decreased due to the COVID-19 restrictions (Gjaka et al., 2021). In addition, the impact of the COVID-19 pandemic on PA level was also investigated among the students’ population specifically, and the results showed a negative impact (Barkley et al., 2020; Galle et al., 2020; Luciano et al., 2020; Grasdalsmoen et al., 2020; Osipov et al., 2021). A previous systematic review was conducted to analyze the impact of the COVID-19 lockdown on PA level of university students, and the results showed a significant reduction in the PA level. However, these results may not be generalizable to other populations, as each country had a different system and level of restrictions during the pandemic (López-Valenciano et al., 2021).

According to the existent literature, the medical students have a high prevalence of depression, anxiety, and psychological distress level than the general population (Dyrbye, Thomas & Shanafelt, 2006). In addition to that, the students might be exposed to higher psychological distress due to the stress caused by the pandemic. The current study aimed to evaluate the impact of the COVID-19 pandemic lockdown on PA levels and other lifestyle aspects (*e.g.*, reducing compliance to exercise and gaining body weight) among the Saudi medical students, and to explore their perspectives toward it. It was hypothesized that the lockdown due to COVID-19 has a negative impact on the Saudi medical students’ PA levels and other aspects of lifestyle.

## Materials and Methods

### Design and selection of study subjects

This was an observational, cross-sectional study using an online survey. Students from various medical sciences fields (applied medical sciences, dental, medicine, nurse, and pharmacy) have been targeted to complete the survey between April 2020 to May 2020 at the time of the lockdown (COVID-19 pandemic) period at King Saud University. At this time the university shifted face-to-face teaching to online E-learning for all courses, theoretical and practical sessions, with the same pre-pandemic teaching timetable. The students were also banned to do any outdoor exercises or activities and were not allowed for them to leave their houses unless for urgent situations such as going to the hospital or buying food with other important stuff during the restricted time that has been scheduled by the Ministry of Health in Saudi Arabia. The study protocol was approved by the Institutional Review Board, University Medical City, King Saud University (Ref. No 21/0356/IRB).

The study included students from various medical sciences fields, who voluntarily agree to join the study and were able to understand and answer the questionnaires. Students who are not registered for courses during this period of time (during data collection) or who have visual or hearing problems were excluded. All participants were non-randomly selected from medical colleges by using a snowball sampling method.

### Instruments and procedures

Through an online survey platform, participants answered an anonymous, confidential online questionnaire in English. An announcement message explaining the study and asking participants whether they accept to participate was sent to participants. If they agree to take part in the study, they are immediately directed to the survey questions’ page on the following page. As social-distancing protocols were put in place and lockdown was enforced during the COVID-19 pandemic, researchers decided that the most practical way to reach target participants was by advertising and disseminating the questionnaire through social networks (such as Facebook, Twitter, and WhatsApp). Additionally, by disseminating surveys *via* social networks, it was possible to contact a wide range of participants from various medical specialties.

The questionnaire consisted of three sections. Section one consisted of the participant’s demographic data which included participants’ age, gender, weight, height, academic program, and academic level. Section two, developed by the study authors and reviewed by a peer reviewer, consisted of five statements related to the impact of the lockdown on PA participation and lifestyle. This section was piloted for clarity before distributing it to the participants. Section three used the International PA Questionnaire—Short Form (IPAQ-SF), to measure the PA level (available online on the IPAQ official website (http://www.ipaq.ki.se)) (Craig et al., 2003). The IPAQ-SF is a self-reported questionnaire that asks participants about the time they spent in the last seven days on various PA intensities (vigorous, moderate, walking) for a minimum of 10 min at a time (Craig et al., 2003). The overall score of PA was calculated using a metabolic equivalent (MET) task scored in minutes/week (MET-min/week) (Craig et al., 2003; Haskell et al., 2007). The PA was reported in this study as a continuous score (https://sites.google.com/site/theipaq/) and a categorical score (inactive (<600 MET-min/week), sufficiently active (≥600 and <3000), and very active (≥3000)).

### Sample size

The sample size was calculated using a web-based calculator (https://www.calculator.net/sample-size-calculator.html). The calculation was performed by setting the statistical power at a confidence level of 95% and margin of error of 5%, and a previously known population size of the medical students in the university (*N* = 6,288). Hence, the size of the sample required for this study was 363 participants.

### Statistical analysis

All statistical analysis was performed using the SPSS statistical software package version 24 (IBM Corp, New York, NY, USA). Descriptive statistical analysis was performed using absolute and relative frequencies for categorical variables and mean with standard deviation (SD) for the quantitative variables. The Kolmogorov–Smirnov test was used to verify the normality of the quantitative variables. To describe the perspectives of participants on the impact of COVID-19 on their lifestyle and PA, the frequency of responses to the statements was reported. Responses of the participants to the five questions were also categorized into two groups according to their total PA (MET-min/week): Group A (strongly agree and agree) and group B (neutral, disagree, and strongly disagree). A *T*-test was used to study the difference between the two groups. The only variable reported with a missing data was the “gender”, and the percentage of missing data before imputation was 10.1%. The missing data was imputed for the “gender” by using the participant’s first name as an indication for their gender. A *p*-value of less than 0.05 has been considered to be statistically significant.

## Results

### Characteristics of the respondents

**Figure 1:**
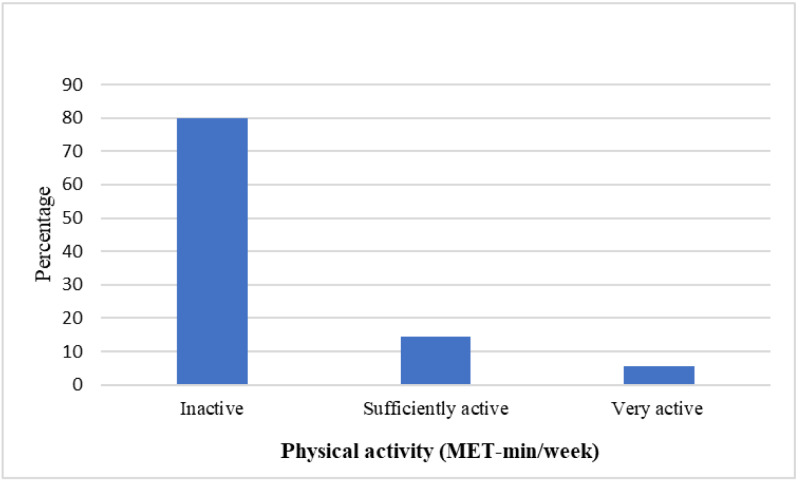
Level of physical activity among the university medical students in Saudi Arabia during the COVID-19 pandemic lockdown. Inactive: reporting < 600 MET-min/week, sufficiently active: reporting ≥ 600 and < 3,000 MET-min/week, and very active: reporting ≥ 3,000 MET-min/week. Abbreviations: MET, metabolic equivalent; min, minutes.

A total of 396 questionnaires were completed and included in this study. The demographics of the included participants, classified by the level of PA, are presented in Table 1. The age average of all participants was 21.1 ± 1.6 years old. Among the 396 questionnaires, there were 253 females (63.9%), and 60.4% of participants were with normal weight. Participants classified as inactive comprised 80.0% of the sample (Fig. 1).

**Table 1:** Demographics characteristics of participants.

		**Physical activity (MET-min/week)**			
Characteristics	All participants (*n* = 396)	Inactive (*n* = 317)	Sufficiently active (*n* = 57)	Very active (*n* = 22)	*P* value^*^
	No. (%)	%	%	%	
**Age (years), mean (SD)**	21.1 (1.6)	21.0 (1.4)	21.3 (2.3)	21.1 (1.7)	0.025
**Sex**					0.985
Male	143 (36.1)	80.4	14.0	5.6	
Female	253 (63.9)	80.1	14.4	5.6	
**BMI, kg/m** ^**2**^					0.327
Underweight (<18.5)	53 (13.4)	88.7	7.5	3.8	
Normal (18.5 to 24.9)	239 (60.4)	76.6	16.3	7.1	
Overweight (25 to 30)	71 (17.9)	83.1	12.7	4.2	
Obese (≥30)	33 (8.3)	84.8	15.2	0.0	
**Specialty**					0.164
Applied Medical Sciences	307 (77.5)	81.1	13.4	5.5	
Dental	11 (2.8)	90.9	9.1	0.0	
Medicine	26 (6.6)	73.1	23.1	3.8	
Nurse	14 (3.5)	71.4	7.1	21.4	
Pharmacy	38 (9.6)	76.3	21.1	2.6	
**Hours per semester**					0.088
12–14	89 (22.5)	87.6	5.6	6.7	
15–17	178 (44.9)	77.5	16.3	6.2	
18–20	129 (32.6)	78.3	17.8	3.9	
**Prevalent chronic diseases**					0.433
No	364 (91.9)	80.8	13.7	5.5	
Yes	32 (8.1)	71.9	21.9	6.3	

**Notes.**

Abbreviations BMI body mass index n number

Inactive: reporting <600 MET-min/week, sufficiently active: reporting ≥ 600 MET-min/week and <3,000, and very active: reporting ≥ 3,000 MET-min/week.

* Chi-squared tests for group differences.

### Perspectives of medical specialties students toward the impact of the lockdown during the COVID-19 pandemic on their lifestyle and PA

The perspectives of students received regarding the PA level reflected the results of the quantitative analysis. Of the medical students who were surveyed, 52.8% “strongly agreed” or “agreed” that they were exercising regularly in the gym before the lockdown. Among those who were exercising, 46.9% “strongly agreed” or “agreed” that the lockdown and transition to online learning affected their compliance to exercise. Furthermore, the majority of participants “strongly disagreed” or “disagreed” that online learning helped them to increase their PA.

We asked medical specialties students whether shifting to online learning had an impact on other aspects of their life (Table 2*).* Approximately, 42.4% of participants reported that online learning made them gain weight. Moreover, 53.6% of participants “strongly agreed” or “agreed” that online learning had a negative impact on their psychological status.

## Discussion

This study aimed to describe the impact of the lockdown on PA levels as well as other aspects of lifestyle among the Saudi students of medical specialties during the lockdown and to describe their perspectives toward it. According to the findings of this study, approximately 80% of students were categorized as inactive. These results were consistent with the results of previous studies conducted on students which reported that during the COVID-19 pandemic the student population showed decreased PA levels (Barkley et al., 2020; Galle et al., 2020; Luciano et al., 2020; Grasdalsmoen et al., 2020; Osipov et al., 2021). Moreover, a previous systematic review that aimed to analyze the impact of COVID-19 lockdown on the PA levels among university students reported a significant reduction of PA levels (López-Valenciano et al., 2021).

**Table 2:** Perspectives of medical students toward the impact of the lockdown during COVID-19 pandemic on their physical activity level and lifestyle.

		Responses, n (%)				
		Strongly disagree	Disagree	Neutral	Agree	Strongly agree
S1	Before the lockdown and the transition to online learning I was exercising in the gym regularly	77 (19.4)	27 (6.8)	83 (21.0)	107 (27.0)	102 (25.8)
S2	The lockdown and the transition to online learning affect my compliance to exercise	39 (9.8)	58 (14.6)	113 (28.5)	117 (29.5)	69 (17.4)
S3	I think online learning has a positive effect on my physical activity level	110 (27.8)	103 (26.0)	73 (18.4)	71 (17.9)	39 (9.8)
S4	I have gained weight after the transition to online learning	63 (15.9)	89 (22.5)	76 (19.2)	90 (22.7)	78 (19.7)
S5	I think online learning has a negative effect on my psychological status	38 (9.6)	64 (16.2)	82 (20.7)	108 (27.3)	104 (26.3)

A recent study conducted among health college students reported a lower percentage compared to our results, which was approximately 63% of students classified as physically inactive (Mahfouz et al., 2020). The resulting home restraint due to COVID-19 may have limited students’ abilities to attend their classes as well as reduced their access to community and environments in general. Lack of time and academic pressure might also be considered as possible reasons for the decreased PA levels (Zain et al., 2021). This is a problematic as physical inactivity was reported as a public concern among the Saudi Arabian population before the pandemic and people may become less and less active during the pandemic (Al-Hazzaa, 2018). Physical inactivity is a major public concern with a substantial impact on both individual and socioeconomic levels (Grasdalsmoen et al., 2020). The findings of the current study have important public health implications. As such and in response to the Saudi vision 2030, universities should consider promoting their students to take part in sports and physical activities and integrate this into the university environment (Grasdalsmoen et al., 2020). For example, PA-based interventions using active videogames might be of more interest to students and seem to be enjoyable and motivating (Viana & de Lira, 2020).

Perspectives of students toward the impact of the lockdown on their PA level and other lifestyle aspects were also explored. There were 53% of students agreed that they were exercising regularly in the gym before the lockdown. However, approximately 47% of students agreed that the lockdown and the transition to online learning impacted their compliance to exercise. This result agrees with a previous study conducted on university students which revealed that PA level decreased, and the sedentary behavior increased significantly during the pandemic period compared to the pre-pandemic period (Jalal et al., 2021). Furthermore, our results found that students perceived that online learning did not help them to increase their PA levels. This might be explained by the fact that students did not move to and from classes or clinical visits which might have decreased the PA levels even further (Luciano et al., 2020).

Nearly all the of the students’ perspectives toward their lifestyle showed the influence of shifting to online learning on their weight. Approximately, 42% of participants agreed that online learning made them gain weight. This might be as a result of the decreased amount of PA and a worsening diet. A previous study demonstrated that the meal patterns and types of food were unhealthier during the confinement period (Ammar et al., 2020).

Moreover, 54% of participants agreed that online learning had a negative impact on their psychological status. Our results are consistent with a previous study which found that moderate-high level of impact of COVID-19 was associated with a higher psychological distress and lower health-related quality of life (Alzahrani et al., 2021). However, the study of Alzahrani et al. has demonstrated that the relationship between COVID-19 impact and psychological distress was moderated by the PA level; where psychological distress for people reporting high-level impact was lower in participants who met PA guidelines than those who did not. Furthermore, moderate and high levels of PA, were protective factors against anxiety or depression among the Chinese college students (Xiang et al., 2020). Hence, current PA recommendations should be promoted during future pandemics to enhance healthy lifestyles.

The current study has few strengths. This study reported a higher level of physical inactivity levels among the Saudi medical students compared to previous findings which needs a call for action. Further, this study reported the perspectives of medical students on how these high levels of physical inactivity impacted some aspects of their lifestyle negatively (*i.e.,* compliance with exercise and gaining more weight). However, this study has also some limitations. One major limitation is that PA level was measured using a self-report questionnaire which might result in a bias and could cause inaccurate estimation of PA amount and consequently misclassification of PA level. Nevertheless, this inaccurate estimation might be decreased to some degree by the convergent validity shown by the IPAQ questionnaire when it is compared with the accelerometery device in the measuring of PA.

## Conclusions

This study found that approximately 80% of students were categorized as inactive during the COVID-19 pandemic concurring with lockdown measures. This study also reported the perspectives of the medical students about the negative influence of the lockdown on their PA level and other lifestyle aspects, for example lower compliance to their exercise and gaining more weight. This study suggests that there is a need to promote PA in the universities context to improve students’ wellbeing during pandemics.

##  Supplemental Information

10.7717/peerj.14725/supp-1 Supplemental Information 1 Raw data

## Additional Information and Declarations

### Data Availability

The following information was supplied regarding data availability: The raw data are available as a Supplementary File.
